# The A756T Mutation of the *ERG11* Gene Associated With Resistance to Itraconazole in *Candida Krusei* Isolated From Mycotic Mastitis of Cows

**DOI:** 10.3389/fvets.2021.634286

**Published:** 2021-08-11

**Authors:** Jun Du, Wenshuang Ma, Jiaqi Fan, Xiaoming Liu, Yujiong Wang, Xuezhang Zhou

**Affiliations:** ^1^Key Laboratory of the Ministry of Education for the Conservation and Utilization of Special Biological Resources of Western China, Ningxia University, Yinchuan, China; ^2^College of Life Science, Ningxia University, Yinchuan, China

**Keywords:** *Candida krusei*, resistance, mycotic mastitis, cows, *ERG11*, *ABC1*, *ABC2*

## Abstract

*Candida krusei* (*C. krusei*) has been recently recognized as an important pathogen involved in mycotic mastitis of cows. The phenotypic and molecular characteristics of 15 *C. krusei* clinical isolates collected from cows with clinical mastitis in three herds of Yinchuan, Ningxia, were identified by matrix-assisted laser desorption ionization–time of flight mass spectrometry. In addition to sequencing analysis, the *ERG11* gene that encodes 14α-demethylases, the expression of the *ERG11* gene, and efflux transporters *ABC1* and *ABC2* in itraconazole-susceptible (S), itraconazole-susceptible dose dependent (SDD), and itraconazole-resistant (R) *C. krusei* isolates was also quantified by a quantitative real-time reverse transcription polymerase chain reaction (qRT-PCR) assay. Sequencing analysis revealed three synonymous codon substitutions of the *ERG11* gene including T939C, A756T, and T642C in these *C. krusei* clinical isolates. Among them, T642C and T939C mutations were detected in itraconazole-resistant and -susceptible *C. krusei* isolates, but the A756T substitution was found only in itraconazole-resistant isolates. Importantly, the expression of the *ERG11* gene in itraconazole-resistant isolates was significantly higher compared with itraconazole-SDD and itraconazole-susceptible isolates (*p* = 0.052 and *p* = 0.012, respectively), as determined by the qRT-PCR assay. Interestingly, the expression of the *ABC2* gene was also significantly higher in itraconazole-resistant isolates relative to the itraconazole-SDD and itraconazole-susceptible strains. Notably, the expression of *ERG11* was positively associated with resistance to itraconazole (*p* = 0.4177 in SDD compared with S, *p* = 0.0107 in SDD with R, and *p* = 0.0035 in S with R, respectively). These data demonstrated that mutations of the *ERG11* gene were involved in drug resistance in *C. krusei*. The A756T synonymous codon substitution of the *ERG11* gene was correlated with an increased expression of drug-resistant genes including *ERG11* and *ABC2* in itraconazole-resistant *C. krusei* isolates examined in this study.

## Introduction

Cow mastitis has a major negative impact on dairy industries, causing significant economic losses to farmers. Pathogenically, a wide variety of microorganisms have been identified as causative agents of cow mastitis, mainly bacteria and fungi (1). In cases of fungal infection of the mammary gland, yeasts of *Candida* genus are the most reported fungal pathogens in cow mastitis (2).

Historically, the mycotic mastitis caused by fungi of the *Candida* genus was first described by Fleischer as early as 1930 (3). Although the infection of *C. albicans* was considered as the most common cause of mastitis, cases of cow mastitis caused by the infection of non-albicans *Candida* spp. (NAC), such as *C. krusei, C. parapsilosis, C. glabrata*, and *C. tropicalis*, have increased significantly during the last decade (4). Among these NACs, *C. krusei* ranked as the fifth most common cause of cow mycotic mastitis (5, 6). *C. krusei* was reported as the causative agent of bovine mastitis and bronchopneumonia in Canada, Mexico, Japan, the United Kingdom, Turkey, Poland, and Algeria (7, 8). Our previous investigation also suggested that *C. krusei* was one of the most important pathogens in mycotic mastitis in dairy farms of the Yinchuan region in Ningxia, China. This result was in accordance with the report of Erbaş et al. (9).

*Candida krusei* has been regarded as a multidrug-resistant fungal pathogen because of its intrinsic resistance to fluconazole (FLC) (10, 11), with more than 96% of human clinical and veterinary isolates being fluconazole-resistant (12). Azole is one of the most common antifungal drugs in agricultural practices (13), including itraconazole, ketoconazole, and tetraconazole. Although it has been reported that multiple mechanisms are involved in drug resistance in *Candida* spp., mechanisms involved in alterations of target enzymes and upregulation of multidrug resistance (MDR)-related proteins are the common mechanisms of *Candida* resistant to azoles. In this regard, 14α-lanosterol demethylase (14-DM) is the target enzyme of azoles, which is responsible for the production of an ergosterol precursor and is encoded by the *ERG11* gene. In *C. albicans* and *C. parapsilosis*, the efflux pump genes *CDR1, CDR2*, and *MDR1* are also associated with azole resistance (14). Nowadays, although transporter genes *ABC1* and *ABC2* were involved in drug resistance in *C. krusei* (15, 16), increased lines of evidence suggested that changes in the expression of activity of target enzyme and upregulation of *MDR* were the main mechanisms of drug resistance in *C. krusei* (11, 16). To date, the study on mechanisms of *Candida* in azole resistance has mainly focused on *C. albicans, C. glabrata*, and *C. tropicalis*, but studies on azole resistance mechanisms in *C. krusei*, especially the *C. krusei* isolates from cow mastitis resistant to itraconazole, are limited, and the involvement of *ERG11, ABC1*, and *ABC2* genes in the drug resistance of *C. krusei* has not been determined (17).

In the present study, we evaluated the profile of the susceptibility of *C. krusei* to itraconazole and investigated the potential alterations of the *ERG11* gene and the differential expression of *ERG11, ABC1*, and *ABC2* genes of 15 clinical *C. krusei* isolates that were isolated from cow mastitis in Yinchuan, Ningxia, China.

## Materials and Methods

### Isolation and Identification of Fungal Pathogens

This study was submitted to and approved by the Ethic Committee of Animal Study in Ningxia University. A total of 465 quarter-milk samples were collected from the cows with clinical or subclinical mastitis, which originated from three herds in Yinchuan, Ningxia, China. Clinical or subclinical mastitis was defined by swelling, reduced milk flow, and abnormal milk appearance (watery to viscous with clots varying from gray-white to yellowish). Additionally, other signs of infection such as fever, inappetence, ataxia, and depression were also considered. These cows have been treated with antibiotics before sample collection. The isolates of *C. krusei* were identified by using *Candida* chromogenic medium (CHROMagar, France) (18) and matrix-assisted laser desorption ionization–time of flight mass spectrometry (MALDI-TOF MS) with score values of >2.000 (VITEK® MS, BioMerieux, France) (19) and stored in liquid nitrogen.

### Antimicrobial Susceptibility Tests

The susceptibility assay was conducted using the Clinical and Laboratory Standard Institute (CLSI) broth microdilution (BMD) method. The CLSI BMD method was performed in a 96-well polystyrene microtiter plate in accordance with CLSI M27-A3 and M27-S4 guidelines (20, 21). The ranges of concentrations of tested drugs were as follows: 5-flucytosine (0.03–64 μg/ml), amphotericin B (0.008–16 μg/ml), fluconazole (0.03–64 μg/ml), itraconazole (0.03–64 μg/ml), and ketoconazole (0.03–64 μg/ml) (15, 22). The antifungal drugs were all purchased from Meilun Biotechnologies (Dalian, China). The *C. krusei* NCCLS reference strain ATCC 6258 served as quality control to ensure the test (3, 23); 1 ×10^3^ CFU/ml working suspension of the *C. krusei* isolates was added into the each well. Results were recorded as resistant, susceptible dose dependent, and sensitive as shown in Table 1.

**Table 1:** Minimal inhibitory concentration (MIC) and susceptibility profile of *C. krusei* clinical isolates (*n* = 15).

**Name**	**Susceptibility profile**				
	**ITR**	**AMB**	**FLC**	**5-FC**	**KET**
ATCC 6258	0.06/S	0.5/S	8/S	4/S	0.125/S
CK1	4/R	2/SDD	64/R	8/SDD	1/R
CK2	8/R	4/R	64/R	32/R	1/R
CK3	0.25/SDD	2/SDD	16/SDD	32/R	1/R
CK4	0.25/SDD	2/SDD	64/R	8/SDD	0.25/SDD
CK5	8/R	4/R	64/R	32/R	1/R
CK6	0.5/SDD	2/SDD	64/R	32/R	1/R
CK7	0.06/S	2/SDD	16/SDD	8/SDD	0.25/SDD
CK8	0.25/SDD	2/SDD	64/R	32/R	1/R
CK9	4/R	2/SDD	64/R	32/R	1/R
CK10	0.25/SDD	2/SDD	32/SDD	32/R	1/R
CK11	0.25/SDD	2/SDD	64/R	16/SDD	0.5/SDD
CK12	4/R	4/R	64/R	32/R	1/R
CK13	0.5/SDD	2/SDD	64/R	32/R	1/R
CK14	0.06/S	2/SDD	16/SDD	32/R	0.5/SDD
CK15	0.25/SDD	2/SDD	64/R	16/SDD	1/R

*CK, Candida krusei; S, susceptible; SDD, susceptible dose dependent; R, resistant; 5-FC, 5-flucytosine, MIC break point: S, ≤ 4 μg/ml; SDD, 8–16 μg/ml; R, ≥32 μg/ml; AMB, amphotericin B, MIC break point: S, ≤ 1 μg/ml; SDD, 2 μg/ml; R, >2 μg/ml; FLC, fluconazole, MIC break point: S, ≤ 8 μg/ml; SDD, 16–32 μg/ml; R, ≥64 μg/ml; ITR, itraconazole, MIC break point: S, ≤ 0.125 μg/ml; SDD, 0.25–0.5 μg/ml; R, ≥1 μg/ml; KET, ketoconazole, MIC break point: S, ≤ 0.125 μg/ml; SDD, 0.25–0.5 μg/ml; R, ≥1 μg/ml*.

### PCR Amplification and Sequencing Alignment of the *ERG11* Gene

*Candida krusei* isolates were subcultured twice on Sabouraud agar at 37°C for 18–24 h to revive and ensure the purity of cultures. A single colony was then transferred to 20 ml of liquid YPD (yeast extract 1%, dextrose 2%, and peptone 2%) broth and cultured at 35°C in a shaking incubator (120 rpm) exponential growth phase. The bacteria cells were collected by centrifugation at 3,000 rpm for 20 min, and the bacteria pellet was used for total genomic DNA preparation using a DNI5-A new Plant Genomic DNA Rapid Extraction Kit (Aidlab Biotechnologies, Beijing, China) according to the manufacturer's instruction. The isolated DNA was used as a template for amplification of the *ERG11* gene. The primer set of *ERG11* was designed by Primer 5.0 and synthesized at Sangon Biotech (Shanghai, China), based on the available sequence information of the *C. krusei ERG11* gene (Gene accession number DQ903905) at the National Center for Biotechnology Information (NCBI) (Table 2). The PCR amplification was conducted in 25 μl volume containing 1 μl of genomic DNA (200 ng/μl), 0.5 μl of specific forward and reverse primers (50 μmol/L), and 12.5 μl of 2 × Phanta Max Master Mix (Vazyme Biotech, Nanjing, China). The PCR parameters were set as denaturation for 3 min at 95°C, followed by 35 cycles of 95°C for 15 s, 60°C for 15 s, and 72°C for 1 min, and a final step of elongation (72°C for 5 min). The resultant PCR product was cleaned by gel purification in 1.5% agarose prior to being cloned into the pTOPO-TA Vector using a CV16-Zero Background pTOPO-Blunt Cloning Kit with Blue/white selection (Aidlab Biotechnologies, Beijing, China). The white colonies were analyzed for clones containing the DNA fragment of gene of interest. Eight to fifteen plasmids from clones generated from an identical PCR product were further sequenced for the *ERG11* gene (Sangon Biotech, Shanghai, China). The sequences were then aligned with the online published sequence of the *ERG11* gene of *C. krusei* strain (Gene Accession Number DQ903905) to determine gene mutation (17).

**Table 2:** Primers used in this study.

**Gene**	**Primer sequence^a^(5^**′**^-3^**′**^)^a^**	**Annealing temperature**	**Accession number**	**PCR product size (bp)**
**Sequencing primers**				
ERG11	F: ATGTCCGTCATCAAGGCAAT	60°C	DQ903905	1,587
	R: CTAGTTCTTTTGTCTTCCCTCCC			
**Real-time PCR primers**				
ABC1	F: GATAACCATTTCCCACATTTGAGT	60°C	DQ903907.1	139
	R: CATATGTTGCCATGTACACTTCTG			
ABC2	F: CCTTTTGTTCAGTGCCAGATTG	60°C	AF250037.1	133
	R: GTAACCAGGGACACCAGCAA			
ERG11	F: AGCAACAACAATGTCCGTCA	60°C	DQ903905	108
	R: TTTGTCTTCCCTCCCACTTG			
GAPDH	F: GTGCCAAAAAAGTTATCATC	60°C	CP039612.1	112
	R: AGTTCTACCACCTCTCCAGT			

a *F, forward; R, reverse*.

### Quantitative Real-Time PCR Analysis

For quantitative real-time PCR (qRT-PCR) analysis, total RNA was extracted from *C. krusei* cultures with RNAiso Reagent (TaKaRa, Dalian, China) and reverse transcribed to cDNA with HiScript III RT SuperMix for qPCR (+gDNA wiper) (Vazyme Biotech, Nanjing, China) according to the manufacturer's instruction. For the *ERG11* target gene and GAPDH reference gene, primer pairs were designed with the Primer 5.0 program and synthesized by Sangon, Shanghai, China (Table 2). qRT-PCR was conducted with a 20-μl volume containing the following reagents: 10 μl of 2 × ChamQ Universal SYBR qPCR Master Mix (Vazyme), 1 μl of total RNA sample, 0.5 μl of each primer pair at a concentration of 10 μmol/L, and 8 μl of distilled water. Each reaction was run in triplicate. Samples were subjected to an initial step at 95°C for 3 min, followed by 40 cycles, each of which consisted of 10 s at 95°C and 30 s at 60°C. Melting curves were recorded every 5 s. The fluorescence data were collected and analyzed with the QuantStudio Design Analysis Software 1.3.1. A 2^−Δ*ΔCt*^ algorithm was employed to analyze the relative expression levels of drug-resistant genes at resistant, susceptible dose-dependent, and sensitive strains.

### Statistical Analysis

Statistical analysis was performed with the GraphPad Prism program (GraphPad 8.0.1 Software Inc., San Diego, CA). The two-tailed Student's *t*-test was used to analyze significant differences between gene expression displayed by the distinct *C. krusei* strains; *p* < 0.05 was considered statistically significant.

## Results

### Resistance of *C. krusei* Isolates to Antifungal Agents

A total of 15 *C. krusei* isolates (designated as CK1–CK15) were isolated from clinical samples from April 2018 to October 2019 in three herds at Yinchuan, Ningxia, China. Drug sensitivity testing was performed according to the broth microdilution method M27-A2 (NCCLS 2002), and the result showed that among 15 *C. krusei* isolates, 73.4, 73.4, and 66.7% were resistant to fluconazole (FLC), ketoconazole (KET), and 5-flucytosine (5-FC), respectively. However, 20 and 33.3% of the isolates were susceptible to amphotericin B (AMB) and itraconazole (ITR), respectively. Interestingly, isolates CK2, CK5, CK6, CK8, CK9, CK12, and CK13 showed resistance to both 5-FC and FLC. The rates of resistance to azole of these *C. krusei* isolates are listed in Table 3. Moreover, isolates CK2, CK5, and CK12 showed multidrug resistance to AMB, 5-FC, FLC, KET, and ITR. In contrast, the reference strain ATCC 6258 was susceptible to all of the five antifungal agents. Overall, among these 15 isolates, 5 were isolates resistant to ITR, 8 belonged to susceptible dose-dependent isolates, and 2 were isolates susceptible to ITR (Tables 1, 3).

**Table 3:** Results of antimicrobial susceptibility tests of *C*. *krusei* isolates (*n* = 15).

**Antibiotic**	**Resistant, % (no.)**	**Susceptible dose dependent, SDD, % (no.)**	**Susceptible, % (no.)**
ITR	33.3 (*n* = 5)	53.3 (*n* = 8)	13.3 (*n* = 2)
AMB	20 (*n* = 3)	80 (*n* = 12)	0
FLC	73.4 (*n* = 13)	26.6 (*n* = 4)	0
5-FC	66.7 (*n* = 10)	33.3 (*n* = 5)	0
KET	73.4 (*n* = 13)	26.6 (*n* = 4)	0

### Mutational Analysis in *ERG11* of *C. krusei* Isolates

The *ERG11* gene fragment was amplified from ATCC 6258 and all 15 *C. krusei* isolates. The PCR product of the open frame of the *ERG11* gene was 1,587 bp, which encodes 529 amino acids. Sequencing analysis identified four different mutations, three synonymous mutations (C642T, A756T, and T939C), and one missense mutation (C44T) in these 15 *C. krusei* isolates (Table 4). Synonymous mutations C642T and T939C were presented in all sequenced isolates, but the synonymous mutation A756T was found in the *ERG11* gene of *C. krusei* isolates resistant to itraconazole (Table 4). One missense mutation was also found at 44 bp (C → T) of the *ERG11* gene (Table 4), which resulted in an amino acid alteration from alanine to valine. However, such a missense mutation was also found in the reference strain ATCC 6258 and all *C. krusei* strains, which indicated that the C44T missense mutation might not be associated with drug resistance to azoles in *C. krusei*.

**Table 4:** *ERG11* gene point mutations in *C. krusei* clinical isolates.

**Information of strains**		**ERG11 gene mutation sites**			
**Name**	**ITR susceptibility category**	**44**	**642**	**756**	**939**
DQ903905		T	T	A	T
ATCC 6258	S	C	C	—	—
CK1	R	C	—	T	C
CK2	R	—	—	T	C
CK3	SDD	C	—	—	—
CK4	SDD	—	—	—	C
CK5	R	—	C	T	C
CK6	SDD	C	—	—	—
CK7	S	C	—	—	—
CK8	SDD	—	—	—	C
CK9	R	—	—	T	—
CK10	SDD	C	C	—	—
CK11	SDD	—	—	—	—
CK12	R	—	—	T	C
CK13	SDD	C	—	—	—
CK14	S	—	C	—	C
CK15	SDD	C	—	—	—

*The numbering of the nucleotides shown in this table starts with 1 for the A of the ATG start codon. DQ903905, GenBank Accession no. of C. krusei whose ERG11 sequence published online is used to align with C. krusei clinical isolates in this study*.

### Increased *ERG11* Gene Transcript in Itraconazole-Resistant *C. krusei*

**Figure 1:**
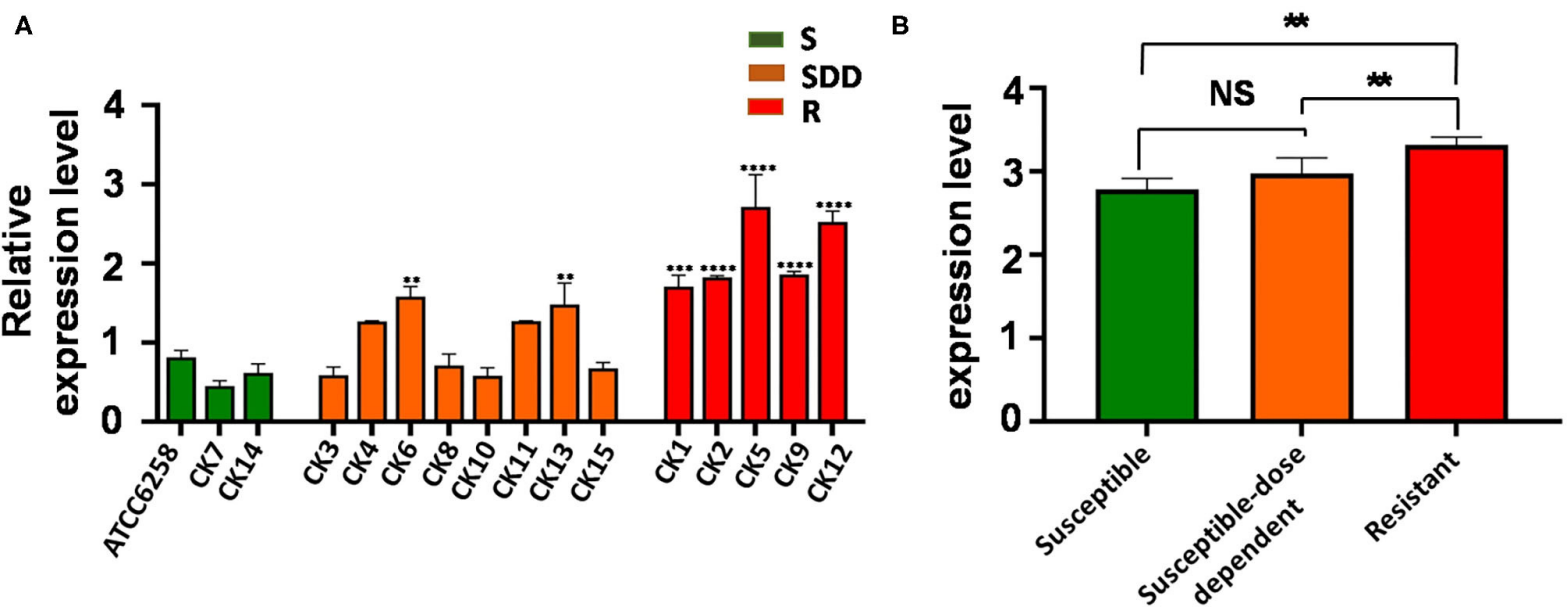
*ERG11* relative gene expression levels in three groups of *C. krusei* clinical isolates. **(A)** Relative levels of *ERG11* mRNA in all the *C. krusei* clinical isolates. *ERG11* gene expression levels were quantified and normalized relative to the reference gene, GAPDH; S, itraconazole-susceptible; SDD, itraconazole-susceptible dose dependent; R, itraconazole-resistant. Asterisks indicate that the difference between field isolates with the reference strain ATCC 6258 is significant compared to the reference strain ATCC 6258: **p < 0.01 in CK6, CK13 with ATCC6258; ***p < 0.001 in CK1 with ATCC6258; and ****p < 0.0001 in CK2, CK5, CK9, CK12 with ATCC6258. **(B)** Log10+3 fold increase of gene expression levels in three groups (NS, no significance in SDD compared with S; *p < 0.05 in R with SDD; **p < 0.01 in R with S).

In order to examine whether an alteration of *ERG11* gene expression was correlated with the drug resistance of *C. krusei* clinical isolates, the transcript of the *ERG11* gene was accessed by a qRT-PCR assay. In comparison with the reference strain ATCC 6258, the relative *ERG11* gene expression of field isolates in five itraconazole-resistant isolates was significantly upregulated (*p* < 0.01), while only two itraconazole-susceptible dose-dependent isolates showed a significantly upregulated *ERG11* gene expression (*p* < 0.01, Figure 1A). The result showed that the transcript of the *ERG11* gene in itraconazole-resistant isolates was significantly more abundant than itraconazole-susceptible strains (*p* = 0.0012, Figure 1B) and itraconazole-susceptible dose-dependent (SDD) strains (*p* = 0.0052, Figure 1B). However, there was no significant difference between itraconazole-susceptible dose-dependent isolates and itraconazole-susceptible isolates (*p* = 0.2562, Figure 1B).

### Increased *ABC2* Gene Transcript in Itraconazole-Resistant *C. krusei*

**Figure 2:**
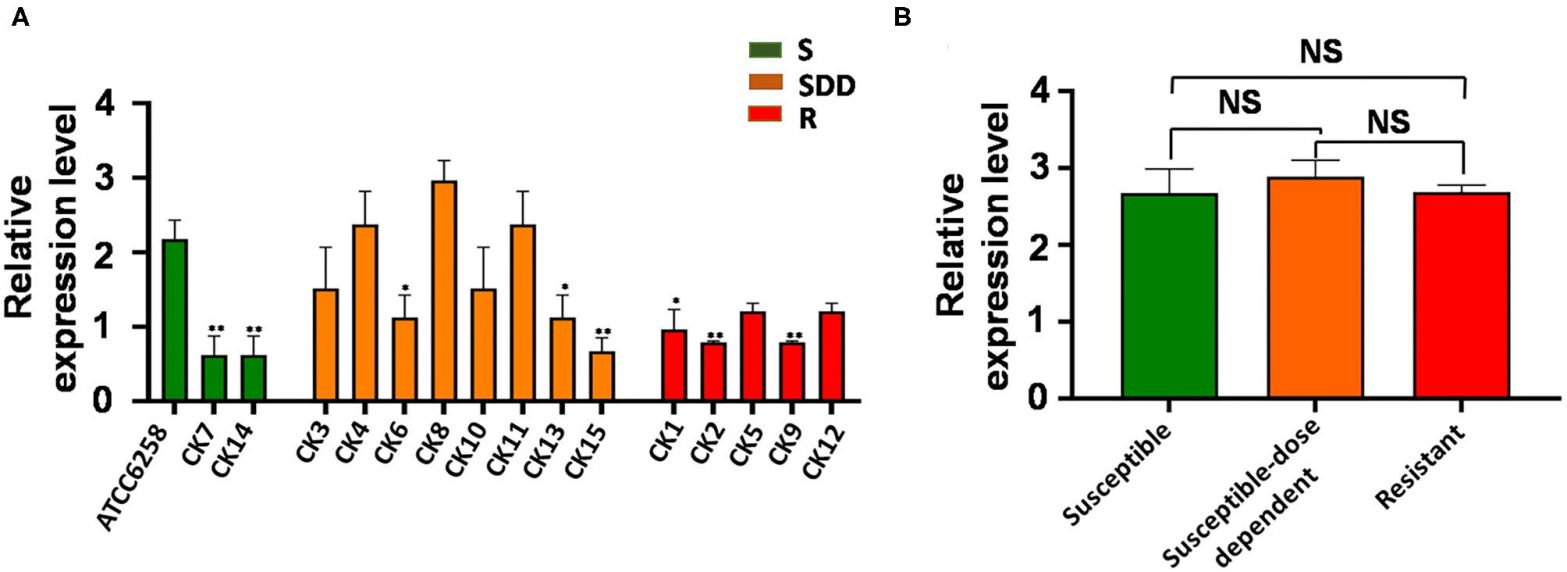
*ABC1* relative gene expression levels in three groups of *C. krusei* clinical isolates. **(A)** Relative levels of *ABC1* mRNA in all the *C. krusei* clinical isolates. *ABC1* gene expression levels were quantified and normalized relative to the reference gene, GAPDH; S, itraconazole-susceptible; SDD, itraconazole-susceptible dose dependent; R, itraconazole-resistant. Asterisks indicate that the difference between field isolates with the reference strain ATCC 6258 is significant compared to the reference strain ATCC 6258: *p < 0.05 in CK1, CK6, CK13 with ATCC6258 and **p < 0.01 in CK2, CK7, CK9, CK14, CK15 with ATCC6258. **(B)** Log10+3 fold increase of gene expression levels in three groups (NS, no significance in SDD compared with S; *p < 0.05 in R with SDD; **p < 0.01 in R with S).

**Figure 3:**
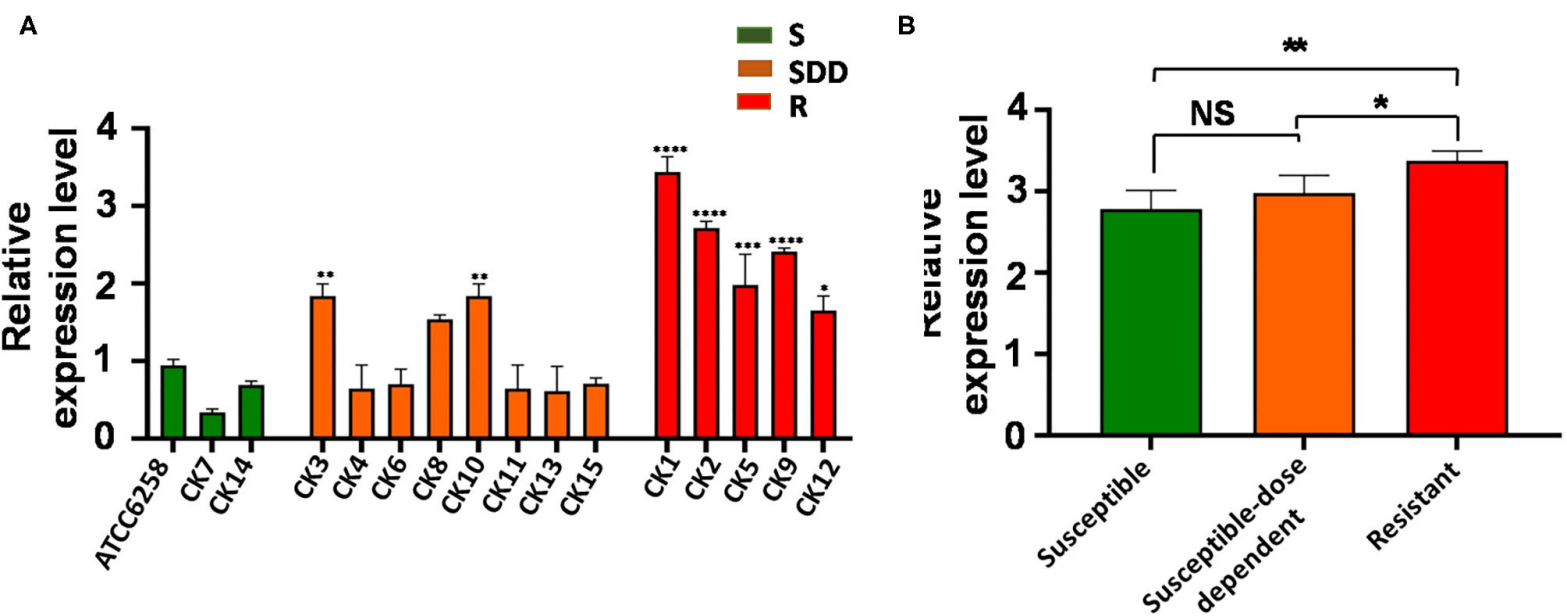
*ABC2* relative gene expression levels in three groups of *C. krusei* clinical isolates. **(A)** Relative levels of *ABC2* mRNA in all the *C. krusei* clinical isolates. *ABC2* gene expression levels were quantified and normalized relative to the reference gene, GAPDH; S, itraconazole-susceptible; SDD, itraconazole-susceptible dose dependent; R, itraconazole-resistant. Asterisks indicate that the difference between field isolates with the reference strain ATCC 6258 is significant compared to the reference strain ATCC 6258: *p < 0.05 in CK12 with ATCC6258; **p < 0.01 in CK3, CK10 with ATCC6258; ***p < 0.001 in CK5 with ATCC6258; and ****p < 0.0001 in CK1, CK2, CK9 with ATCC6258. **(B)** Log10+3 fold increase of gene expression levels in three groups (NS, no significance in SDD compared with S; *p < 0.05 in R with SDD; **p < 0.01 in R with S).

ABC transporters are involved in drug resistance; next, we therefore sought to examine the alteration of ABC transporters in *C. krusei* isolates. Interestingly, unlike the *ERG11* gene, none of field isolates showed an upregulated *ABC1* gene expression as compared with that of the reference strain ATCC 6258 (Figure 2A). The results of qRT-PCR for *ABC1* genes showed that the expression of *ABC1* gene mRNA was not significantly different between itraconazole-resistant isolates, itraconazole-susceptible dose-dependent isolates, and itraconazole-susceptible strains (*p* = 0.3844, *p* = 0.9997, and *p* = 0.2996, respectively, Figure 2B). Similar to that seen in the *ERG11* gene, the relative *ABC2* gene expression was extremely significantly upregulated in four itraconazole-resistant isolates (*p* < 0.01) and significant in one itraconazole-resistant isolate (*p* < 0.05), as compared with the reference strain ATCC 6258, whereas only two itraconazole-susceptible dose-dependent isolates showed an extremely significant upregulation of *ABC2* gene expression compared to the reference strain ATCC 6258 (*p* < 0.01, Figure 3A). Intriguingly, the transcript of the *ABC2* gene in itraconazole-resistant isolates was more abundant relative to itraconazole-susceptible strains (*p* = 0.0035) and itraconazole-susceptible dose-dependent strains (*p* = 0.0107, Figure 3B). No significant difference was found in the expression of the *ABC2* gene was determined between the itraconazole-susceptible dose-dependent group and itraconazole-susceptible *C. krusei* isolates (*p* = 0.4177, Figure 3B).

## Discussion

Our previous study demonstrated that *C. krusei* was a predominant pathogen isolated from mycotic mastitis of cows in Yinchuan, Ningxia, China (12), suggesting that it may be an important fungal pathogen of mycotic mastitis of cows in this area. The crucial roles of drug-resistant genes *ERG11, ABC1*, and *ABC2* in FLC-resistant clinical isolates of *C. krusei* from human have been well-established (15, 24). However, the pathogenic molecular mechanism of *C. krusei* isolated from cow mastitis remains unclear. In the present report, we evaluated the susceptibility profiles and mutations in the *ERG11* gene in 15 clinical *C. krusei* isolates. The expression of drug-resistant genes *ERG11, ABC1*, and *ABC2* between isolates susceptible, susceptible dose-dependent, and resistant to ITR was also analyzed. We identified three synonymous mutations and one missense mutation in the *ERG11* gene in these clinical *C. krusei* isolates, as previously described in human *C. krusei*. Furthermore, the A756T was only presented in ITR-resistant strains, suggesting that it might be correlated with drug resistance in *C. krusei*, while mutations T642C and T939C were presented in all these 15 *C. krusei* isolates. The expression of drug-resistant *ERG11* and *ABC2* was also significantly higher in ITR-resistant *C. krusei* isolates compared to ITR-susceptible and susceptible dose-dependent isolates, suggesting a correlation of mutations of the *ERG11* gene with the resistance to antifungal agents in *C. krusei*.

In the present study, based on the CLSI BMD method's susceptibility to ITR, the 15 *C. krusei* clinical isolates from cows with clinical mastitis could be divided into three groups: ITR-susceptible (2 isolates), ITR-susceptible dose-dependent (8 isolates), and ITR-resistant (5 isolates). The antifungal testing showed that 13.3, 53.3, and 33.3% were susceptible, susceptible dose-dependent, and resistant to itraconazole among these *C. krusei* clinical isolates, respectively. Notably, 13 of the 15 *C. krusei* isolates (73.4%) were also resistant to FLC and KET, 10 of the 15 *C. krusei* isolates (66.7%) were resistant to flucytosine, and 3 of the 15 isolates (20%) were resistant to amphotericin B. This finding was consistent with a study by Namvar et al. (25), but was different from reports by others (8, 9, 26). Of interest, the rate of resistance to antifungal agents in these *C. krusei* isolates was lower than our previous findings (12), which might be attributed to the reduction in the use of antifungal drugs during breeding. Consistent with our previous studies, *C. krusei* isolates were double-resistant and multidrug-resistant to antifungal drugs. It is strongly recommended that ketoconazole and other azole antifungal agents should not be used in the treatment of *C. krusei* infection in dairy cows in Ningxia, China, owing to high drug resistance.

It is worth noting that CK2, CK5, and CK12 showed resistance to amphotericin B, but such cases are rare (27). However, several lines of studies evidenced an increased minimum inhibitory concentration (MIC) of amphotericin B in *C. krusei* isolates. In *Candida* spp., resistance to amphotericin B was found to be associated with a decreased ergosterol content of cell membrane (28–30). In addition, an inactivation of *ERG3* could substitute 14α-methylfecosterol with ergosterol, thus reducing ergosterol levels, which, in turn, resulted in deficient ergosterol to counter the function of amphotericin B (31). Moreover, several other mutations in the ergosterol biosynthetic genes such as *ERG2, ERG5, ERG6*, and *ERG24* can also result in *C. albicans* and *C. glabrata* resistant to amphotericin B (32–34). However, such mutations have not been reported in *C. krusei* and further studies are needed in future studies.

A number of studies have focused on *ERG11* gene mutation in *Candida* species (35–37). In order to fully understand *ERG11* gene mutation of *C. krusei*, the whole open reading frame of the *ERG11* gene was amplified for sequencing analysis, and three synonymous mutations and one missense mutation were identified in this study. C44T has one missense mutation and was found in all 15 isolates and the reference strain, suggesting that it had no impact on the itraconazole resistance of *C. krusei*. Molecularly, the C44T mutation resulted in the alteration of alanine to valine in the 15th amino acid of 14α-lanosterol demethylase (14-DM); this mutation might occur outside the active site of the *ERG11* gene, which might not affect the mutual interaction of azole and 14-DM, or a single missense mutation might not be sufficient to change the affinity of the 14-DM to azole (15). Moreover, synonymous mutations (C642T, A756T, and T939C) in the 15 isolates were consistent with a previous report (24). Synonymous mutations can affect transcription, splicing, mRNA transport, and translation, any of which could change phenotype, rendering the synonymous mutation non-silent (38). He et al. (15) also reported that C642T, T1389C, and G1536C mutations occurred in all the experimental strains. In addition, the T1389C mutation was also reported by Ricardo et al. (16). Tavakoli et al. (35) revealed a heterozygous polymorphism at position T939C in the *ERG11* coding region and speculated that this polymorphism might play a key role in the transcriptional regulation of genes and be involved in the processes of ergosterol biosynthesis. Of note, several previously reported mutations, including the T418C missense mutation (16), and C51T, T1389C, and G1536C synonymous mutations (15), have not been found in this study; thus, limited clinical isolates (15) were analyzed. Mechanistically, previous studies on *C. albicans* and *C. tropicalis* have demonstrated that the missense mutation was associated with resistance to azole, which was partly through changing the conformation of the target enzyme 14-DM, which, in turn, decreased its drug affinity and influenced the enzyme's function in ergosterol biosynthesis (39, 40). The resistance mechanism of these resistant strains may be due to one or multiple mutations in these genes, which needs further investigation.

In addition, the expression of *ABC2* and *ERG11* genes was significantly upregulated in *C. krusei* veterinary clinical isolates resistant to itraconazole. Previous studies demonstrated that resistance to azole was also due to the increased expression of *ERG11*. This results in insufficient azole activity owing to the overproduction of the target enzyme (41). Although *ERG11* overexpression has been reported in *C. krusei*, the mechanism behind the overexpression remains unclear (15, 35). Another mechanism of resistance to azole is via the decreased intracellular accumulation of azole. This can be due to efflux pump activity or changes in the cell membrane. In this regard, drug efflux pumps belong to either the ATP-binding cassette (ABC) family of transporters or the major facilitator super family (MFS) class. These proteins can pump out fungicidal compounds across the cell membrane, and their overexpression results in multidrug resistance phenotype in pathogenic fungus (17). In contrast to members of the MFS class that are actuated by electrochemical proton-motive force, ABC family members depend on the hydrolysis of ATP for energy (42, 43). Indeed, along with the decrease of susceptibility to itraconazole, the *ABC2* gene mRNA expression in the isolates appeared to increase. Although it was considered that Abc1p played an important role in the innate FLC resistance of *C. krusei* (16, 17), the *ABC2* gene could be activated slower than the *ABC1* gene in the presence of voriconazole (16). Given voriconazole tolerance, the Abc1p efflux pump is supposed to be more efficient in expelling drug and plays a late role in the development of resistance, and the accumulation of itraconazole in drug-susceptible *C. krusei* was higher than that of resistant strains (44). Interestingly, Venkateswarlu et al. found that two isolates highly resistant to fluconazole showed a different sensitivity to itraconazole, suggesting that itraconazole and fluconazole in *C. krusei* may have different resistance mechanisms (44). The authors stated that *C. krusei* resistant to itraconazole was due to decreased drug accumulation in cells and speculated that there might exist more efflux pumps contributing to itraconazole resistance of *C. krusei*, which could be well-explained in this study. The overexpression of both *ERG11* and *ABC2* has been reported to be involved in itraconazole resistance in *C. krusei* (15, 35). However, an unusual transient or stable resistance of *C. krusei* to voriconazole has also emerged. Overexpression of *ABC2* and *ERG11*, as observed in itraconazole resistance, imparts a transient resistance to voriconazole, while a more stable resistance was observed due to the overexpression of *ABC1* and point mutation in *ERG11* (16). Taking this into account, we speculate that the resistance mechanisms of itraconazole and voriconazole in *C. krusei* clinical isolates may be different. Moreover, other genes encoding ATP-dependent efflux transporters may occur in *C. krusei*, such as a CgSNQ2 homologous gene that was verified as an azole-associated resistance gene in *C. glabrata* (45). Although the *C. krusei* genome has been sequenced, it is not completely annotated yet; thus, other transporter genes were not assessed. Our mRNA expression data showed that Abc2p may play a more important role in itraconazole resistance of *C. krusei*, instead of Abc1p.

This study has enriched our knowledge in the veterinary clinical *C. krusei* resistance gene expression and mutation data by comparing the difference between the veterinary clinical and the human clinical *C. krusei*, and further deepened our understanding of the resistance mechanism of *C. krusei* in veterinary clinics. The limitation of this study is that the sample size was small and no drug susceptibility test and resistance mechanism research related to echinocandin has been included, which require further investigations.

In conclusion, in this study, we found that *C. krusei* veterinary clinical isolates exhibited a different susceptibility to antifungal agents. Mechanistically, the A756T mutation in the *ERG11* gene resulted in an upregulation of drug-resistant genes *ERG11* and *ABC2*, substantially enhancing the resistance to itraconazole of *C. krusei*. Although we have identified four point mutations in the *ERG11* gene associated with itraconazole resistance and have already described their role on the itraconazole resistance of *C. krusei*, it is necessary to confirm the effect of these mutations by site-directed mutagenesis of the *C. krusei* strain in the future. Nevertheless, this study may thus provide an insight into the mechanism of the resistance of *C. krusei* to antifungal agents, which warrants for further investigation.

## Data Availability Statement

The datasets generated for this study are available on request to the corresponding author.

## Ethics Statement

The animal study was reviewed and approved by Ethics Committee of Animal Research of Ningxia University.

## Author Contributions

JD, YW, and XZ conceived and designed the experiments. JD and JF analyzed the data and drafted the manuscript. JD, WM, JF, and XL performed experiments and acquired data. XL and XZ interpreted data and critically revised the manuscript. All authors read and approved the final version of the manuscript.

## Conflict of Interest

The authors declare that the research was conducted in the absence of any commercial or financial relationships that could be construed as a potential conflict of interest.

## Publisher's Note

All claims expressed in this article are solely those of the authors and do not necessarily represent those of their affiliated organizations, or those of the publisher, the editors and the reviewers. Any product that may be evaluated in this article, or claim that may be made by its manufacturer, is not guaranteed or endorsed by the publisher.
